# CD8^+^ T and NK cells characterized by upregulation of NPEPPS and ABHD17A are associated with the co-occurrence of type 2 diabetes and coronary artery disease

**DOI:** 10.3389/fimmu.2024.1267963

**Published:** 2024-02-23

**Authors:** Chenyu Dai, Damu Wang, Qianqian Tao, Ziyi Li, Peng Zhai, Yingying Wang, Mei Hou, Simin Cheng, Wei Qi, Longyi Zheng, Huaifang Yao

**Affiliations:** ^1^ Department of Cadre Cardiology, The First Affiliated Hospital of Anhui University of Chinese Medicine, Hefei, Anhui, China; ^2^ Department of General Surgery, The First Affiliated Hospital of Anhui University of Chinese Medicine, Anhui Academy of Chinese Medicine, Hefei, Anhui, China; ^3^ Department of Biomedical Engineering, Boston University, Boston, MA, United States; ^4^ Anhui Provincial Children’s Hospital, Children’s Hospital of Fudan University, Hefei, Anhui, China; ^5^ Cancer Research Center, The First Affiliated Hospital of University of Science and Technology of China (USTC), Division of Life Sciences and Medicine, University of Science and Technology of China, Hefei, Anhui, China; ^6^ Department of Endocrinology, Changhai Hospital, Naval Medical University, Shanghai, China

**Keywords:** cardiovascular disease, type 2 diabetes, bioinformatics, hub gene, CD8+T cells, NK cells, immune

## Abstract

**Background:**

Coronary artery disease (CAD) and type 2 diabetes mellitus (T2DM) are closely related. The function of immunocytes in the pathogenesis of CAD and T2DM has not been extensively studied. The quantitative bioinformatics analysis of the public RNA sequencing database was applied to study the key genes that mediate both CAD and T2DM. The biological characteristics of associated key genes and mechanism of CD8^+^ T and NK cells in CAD and T2DM are our research focus.

**Methods:**

With expression profiles of GSE66360 and GSE78721 from the Gene Expression Omnibus (GEO) database, we identified core modules associated with gene co-expression relationships and up-regulated genes in CAD and T2DM using Weighted Gene Co-expression Network Analysis (WGCNA) and the ‘limma’ software package. The enriched pathways of the candidate hub genes were then explored using GO, KEGG and GSEA in conjunction with the immune gene set (from the MSigDB database). A diagnostic model was constructed using logistic regression analysis composed of candidate hub genes in CAD and T2DM. Univariate Cox regression analysis revealed hazard ratios (HRs), 95% confidence intervals (CIs), and p-values for candidate hub genes in diagnostic model, while CIBERSORT and immune infiltration were used to assess the immune microenvironment. Finally, monocytes from peripheral blood samples and their immune cell ratios were analyzed by flow cytometry to validate our findings.

**Results:**

Sixteen candidate hub genes were identified as being correlated with immune infiltration. Univariate Cox regression analysis revealed that NPEPPS and ABHD17A were highly correlated with the diagnosis of CAD and T2DM. The results indicate that CD8^+^ T cells (p = 0.04) and NK^bright^ cells (p = 3.7e-3) are significantly higher in healthy controls than in individuals with CAD or CAD combined with T2DM. The bioinformatics results on immune infiltration were well validated by flow cytometry.

**Conclusions:**

A series of bioinformatics studies have shown ABHD17A and NPEPPS as key genes for the co-occurrence of CAD and T2DM. Our study highlights the important effect of CD8^+^ T and NK cells in the pathogenesis of both diseases, indicating that they may serve as viable targets for diagnosis and therapeutic intervention.

## Introduction

Diabetes mellitus (DM) is a chronic metabolic disorder characterized by hyperglycemia resulting from defects in insulin secretion, insulin resistance, or a combination of both (1, 2). This condition can lead to the long-term damage to organs, nervous system, and blood vessels, which further develop into organ dysfunction or even failure (3). The global prevalence of DM is increasing each year, with predictions indicating that the number of individuals with DM will reach 5.92 billion by 2035 (4, 5), over 90% of which will be type 2 diabetes mellitus (T2DM) (6). While T2DM can be effectively controlled clinically with antidiabetic drugs such as metformin and insulin (7, 8), the inherent metabolic abnormalities contribute to a wide range of diseases and predisposes to complications that threaten human health (9, 10). For this reason, most medical research has focused on the relationship between T2DM and other diseases (11–13).

Over the past 15 years, coronary artery disease (CAD) has become the leading cause of death worldwide, accounting for 15 million deaths in 2016 alone (14), and it is the primary reason of morbidity and death rate among individuals with T2DM (15, 16). There is a strong correlation between the occurrence of CAD and T2DM (1, 17). In their analysis of the Framingham Heart Study, Fox et al. reported that for each ten-year expand in the duration of T2DM, the morbidity and death rate of CAD in patients with T2DM increased by 1.38 and 1.86 times, respectively, compared to those without T2DM (18). The primary pathology underlying CAD is atherosclerosis, a chronic inflammatory response leading to plaque formation and can result in stable angina, unstable angina, sudden cardiac death, and myocardial infarction (MI) (19, 20). Immune cells including monocytes, macrophages, endothelial cells, smooth muscle cells and adipocytes are attracted to atherosclerotic plaques and are considered critical determinants of the disease progression (21, 22). The transition from stable plaques to unstable, rupture-prone plaques is associated with an increased number of T cells displaying signs of early activation within the plaque (23–25). Studies have shown that the number of apoptotic NK cells in the peripheral blood of patients with CAD is significantly increased (26, 27), and that the phenotype of CD8^+^ T cells correlates with the rate of disease progression after the onset of T2DM (28–30). Despite the rapidly increasing prevalence of T2DM, which has proved to be a major account of morbidity and death rate in patients with MI (31, 32), research into the associated inflammation and changes in immune cell function between the two conditions is limited. Therefore, it is crucial to investigate the pathogenesis of MI and T2DM as well as determine the mechanisms of inflammation and immune regulation (33).

**Figure 1:**
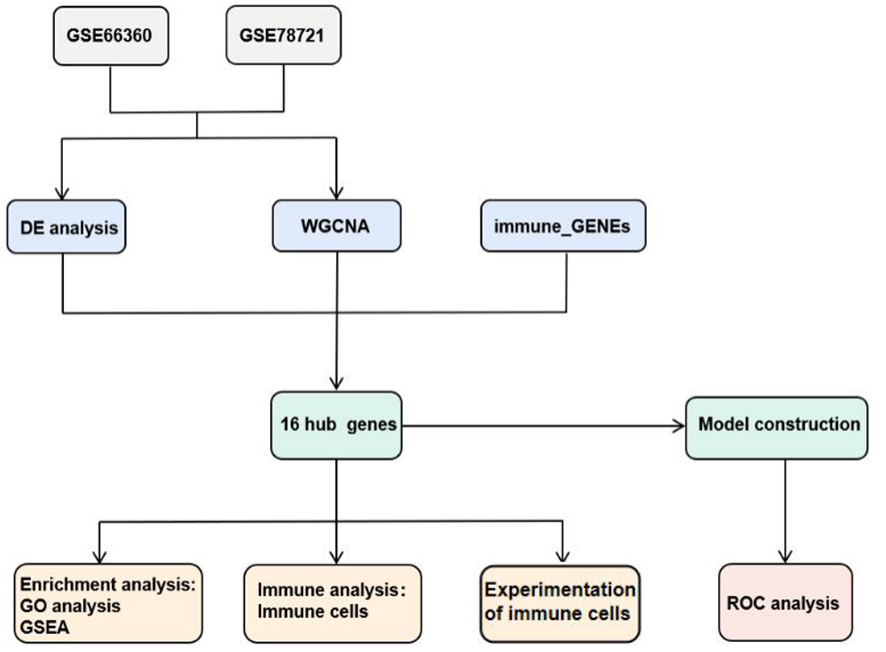
Structure of workflow chart.

Drawing on public data and bioinformatics methods, this study identified 16 candidate hub genes linked with immune genes, MI and T2DM. Gene Ontology(GO) and Gene Set Enrichment Analysis (GSEA) were applied to detailed examine the organic procedure and gateways. Stepwise regression and Univariate Cox regression analysis were accomplished to create diagnostic models of CAD and T2DM and examine sixteen candidate hub genes in diagnostic models, which were authenticated in two specimen datasets, GSE66360 and GSE78721. These models have good potential diagnostic performance value in the clinical diagnosis of CAD and T2DM, and the measurement results of Area Under the Curve values indicated this. As T2DM is an important factor influencing cardiovascular disease and have high correlation with immune cell function. Consequently, we additionally investigated the distribution of immunocytes of specimens and the relevance of various immunocytes with these differentially expressed genes (DEGs), then performed correlation validation analyses of the abnormalities of the above DEGs. For further research, we performed correlation validation analyses of the abnormalities of the above candidate hub genes and immune cells in blood samples from 38 clinical CAD, T2DM patients and 9 healthy individuals. The results indicate a strong association between CAD and the prevalence of CD8^+^ T and NK cells, also suggest the risk of T2DM combined with CAD, providing insights for targeted treatment and control. The article design is shown in **Figure 1**.

## Results

### Identification of CAD and T2DM related gene modules

**Figure 2:**
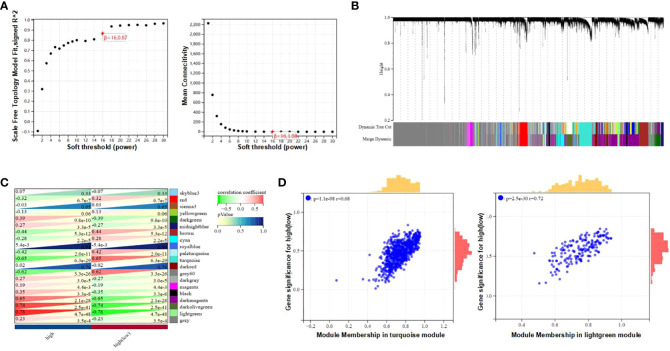
Determination of gene modules related to coronary heart disease. **(A)** Constructing Soft Threshold Power (β) based on dimension independence and mean connectivity. **(B)** Gene block mass related to CAD are displayed in various colors under the gather dendrogram. **(C)** The correlation between gene modules and CAD is depicted through heat maps. The upper left side displays the coefficient of correlation, and the lower right side displays the P-value. **(D)** The correlation between the most positively correlated and negatively correlated modules in CAD, different member relationships, and gene conspicuousness.

The Weighted Gene Co-expression Network Analysis (WGCNA) was employed to identify interconnected gene clusters or modules with a close relationship to CAD. The most suitable soft threshold power, β = 16.087, was selected based on scale independence (R^2 = 0.87) and mean connectivity (the minimum of about 0) (**Figure 2A**). Following this, module merging was performed, resulting in 20 gene co-expression modules related to CAD, each represented by a different color (**Figures 2B, C**). These colors depict the relationship between the modules and CAD, with turquoise indicating the most positive correlation (232 genes; [CC] = 0.65; P = 6.3e-29) and light green showing the strongest negative correlation (66 genes; [CC]=-0.78; P=4.7e-48) with T2DM. In addition, significant correlations were observed between both the turquoise (r = 0.68) and light green (r = 0.72) module memberships and gene significance for CAD (**Figure 2D**). Consequently, 298 genes within the turquoise and light green modules, which exhibited the strongest associations with CAD, were chosen for further study. The details of the identification of gene modules associated with T2DM can be found in **Supplementary Figure 1**.

### Identification of hub genes associated with immunological signature genes, CAD, and T2DM

**Figure 3:**
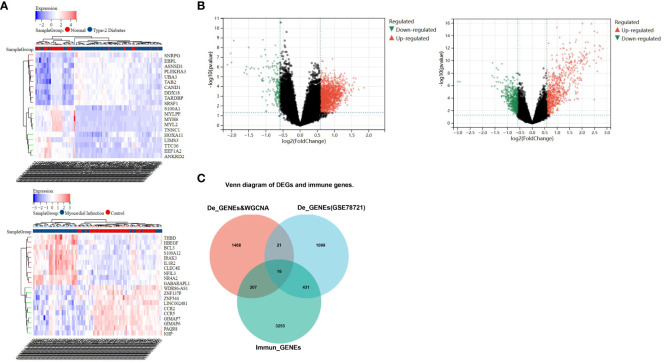
Recognition of differential genes associative with CAD patients and healthy cohorts, T2DM patients and healthy cohorts. **(A)** The top 20 up- and down-regulated differential gene discovered between the CAD and healthy groups, as well as T2DM and healthy groups, are represented by red and blue squares in the heat map, respectively. **(B)** The red and green triangles in the volcano map represent the up- and down- differential genes. **(C)** Venn diagram shows gene intersection of immune gene, the differential genes of T2DM and the differential genes of CAD combined with WGCNA-identified module genes.

In the comparison between the T2DM and normal population, 1567 DEGs were acknowledged, and the comparison between the CAD and healthy control groups, 1414 DEGs were found. These genes were validated using the “limma” package. In the T2DM group, 1384 of these genes were upregulated and 183 were downregulated, while in the CAD group, 815 were upregulated and 599 were downregulated. The top 20 upregulated and downregulated DEGs are depicted in the heatmap (**Figure 3A**), and all DEGs are represented by volcano plots, with red or blue grids reflecting genes which were upregulated and downregulated, separately (**Figure 3B**). Red or green triangles implied genes which were upregulated and downregulated, separately. From the Immunologic Signature gene sets (ImmuneSigDB; MSigDB; Liberzon et al., 2011, Bioinformatics), immune_GENEs were extracted. The ImmuneSigDB contains gene sets representing comprehensive regulatory dynamics of cell types, states, and disturbances within the immune system. These signatures were created through the manual curation of published human immunology studies. Subsequently, an intersection of 298 module genes associated with CAD as identified by WGCNA combined with 1414 DEGs detected by the “limma” package, 3907 immune_GENEs extracted from MSigDB, and 1567 T2DM DEGs associated with T2DM progression led to the selection of 16 candidate hub genes: PI4KA, YWHAZ, ERRFI1, ABHD17, LRRC40, PLSCR4, NPEPPS, SEL1L3, MAP3K2, ZBED5, EIF2B1, CAPN2, ZNF146, BCHE, UQCRC2, USP34. All 16 candidate hub genes are protein-coding and their distribution varies in the human body, mainly concentrated in the brain and lymph nodes (**Figure 3C**; **Table 1**).

**Table 1:** The type and expression of the 16 hub genes.

Genes	Gene type	Expression
PI4KA YWHAZ ERRFI1 ABHD17A LRRC40 PLSCR4 NPEPPS SEL1L3 MAP3K2 ZBED5 EIF2B1 CAPN2 ZNF146 BCHE UQCRC2 USP34	protein coding protein coding protein coding protein coding protein coding protein coding protein coding protein coding protein coding protein coding protein coding protein coding protein coding protein coding protein coding protein coding	Ubiquitous expression in brain (RPKM 52.3), testis (RPKM 23.0) and 24 other tissues Ubiquitous expression in esophagus (RPKM 248.0), brain (RPKM 160.0) and 25 other tissues Broad expression in liver (RPKM 123.7), gall bladder (RPKM 60.1) and 20 other tissues Ubiquitous expression in spleen (RPKM 12.7), bone marrow (RPKM 10.4) and 25 other tissues Ubiquitous expression in brain (RPKM 8.0), testis (RPKM 8.0) and 25 other tissues Ubiquitous expression in fat (RPKM 22.0), gall bladder (RPKM 21.1) and 24 other tissues Ubiquitous expression in esophagus (RPKM 43.2), brain (RPKM 28.6) and 25 other tissues Broad expression in lymph node (RPKM 28.3), stomach (RPKM 23.4) and 20 other tissues Ubiquitous expression in bone marrow (RPKM 11.2), thyroid (RPKM 10.3) and 25 other tissues Ubiquitous expression in lymph node (RPKM 23.1), endometrium (RPKM 21.4) and 25 other tissues Ubiquitous expression in lymph node (RPKM 22.1), skin (RPKM 20.5) and 25 other tissues Ubiquitous expression in lung (RPKM 90.0), gall bladder (RPKM 66.0) and 25 other tissues Ubiquitous expression in thyroid (RPKM 20.8), endometrium (RPKM 19.9) and 25 other tissues Biased expression in liver (RPKM 60.4), brain (RPKM 16.9) and 12 other tissues Ubiquitous expression in heart (RPKM 179.2), duodenum (RPKM 111.5) and 25 other tissues Ubiquitous expression in testis (RPKM 17.6), lymph node (RPKM 17.0) and 25 other tissues

### Functional enrichment analysis and biological process of DEGs

**Figure 4:**
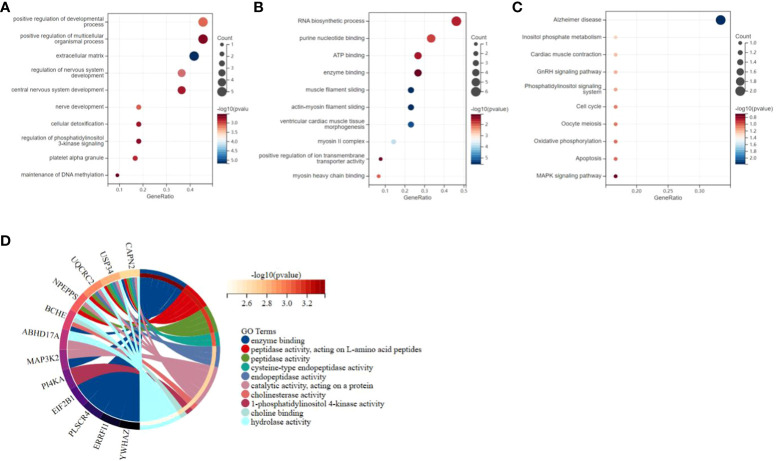
Functional enrichment study of differentially expressed genes and hub genes related to CAD and T2DM progression. **(A–C)** Gene Ontology analysis of CAD and T2DM-associated differential gene. The top 10 enriched Gene Ontology sorts (bioprocess, cell constituent, and molecular function) are revealed. Gene proportions and different ontologies are represented by the X and Y coordinates. The circle extent indicates gene count. **(D)** Kyoto Encyclopedia of Genes and Genomes analysis of the candidate hub genes related to CAD and T2DM progression, immune genes. The left and right portion means the enriched differentially expressed genes and most considerable ontologies, respectively.

GO and Kyoto Encyclopedia of Genes and Genomes (KEGG) annotations were utilized for more detailed biological research of DEGs. GO manifested that the DEGs were chiefly distributed in several biology procedures including 1) platelet alpha granule, maintenance of DNA methylation, positive regulation of multicellular organism process, and regulation of phosphatidylinositol 3-kinase signaling; 2) ATP binding, secretory granule, enzyme binding, and purine nucleotide binding; and 3) Alzheimer’s disease, inositol phosphate metabolism, oxidative phosphorylation, and apoptosis (**Figures 4A–C**). KEGG pathway enrichment analysis demonstrated that these genes were chiefly enriched in endopeptidase activity, hydrolase activity, and cysteine-type endopeptidase activity (**Figure 4D**). GSEA was employed to identify activation pathways in CAD and T2DM, and to distinguish differential regulatory pathways between the high and low expression groups of candidate hub genes. GSEA of hub genes suggested that they were associated with several protein biological processes such as ubiquitin-mediated proteolysis, protein export, and RNA polymerase, neurological related activities like neuroactive ligand-receptor interaction, and other biological processes like olfactory transduction, ubiquitin-mediated proteolysis, nucleotide excision repair, endocytosis, and limonene and pinene degradation (**Supplementary Figure 2**). Among these candidate hub genes, ERRFI1, SEL1L3, ZBED5, ZNF146, ABHD17A, and YWHAZ were implicated in the biological process of protein output. ERRFI1, PI4KA, ZBED5, UQCRC2, and ZNF146 were implicated in the biological process of olfactory transduction. ZNF146 and ABHD17A were implicated in the biological process of the spliceosome. ERRFI1 was implicated in the biological process of RNA polymerase. Among these candidate hub genes, SEL1L3 was involved in most biological pathways, including ubiquitin-mediated proteolysis, protein export, pyrimidine metabolism, nucleotide excision repair, pathogenic Escherichia coli infection, and neuroactive ligand-receptor interaction. In contrast, PLSCR4 was only involved in the biological process of limonene and pinene degradation.

### Construction and validation of diagnostic models and hub genes

**Figure 5:**
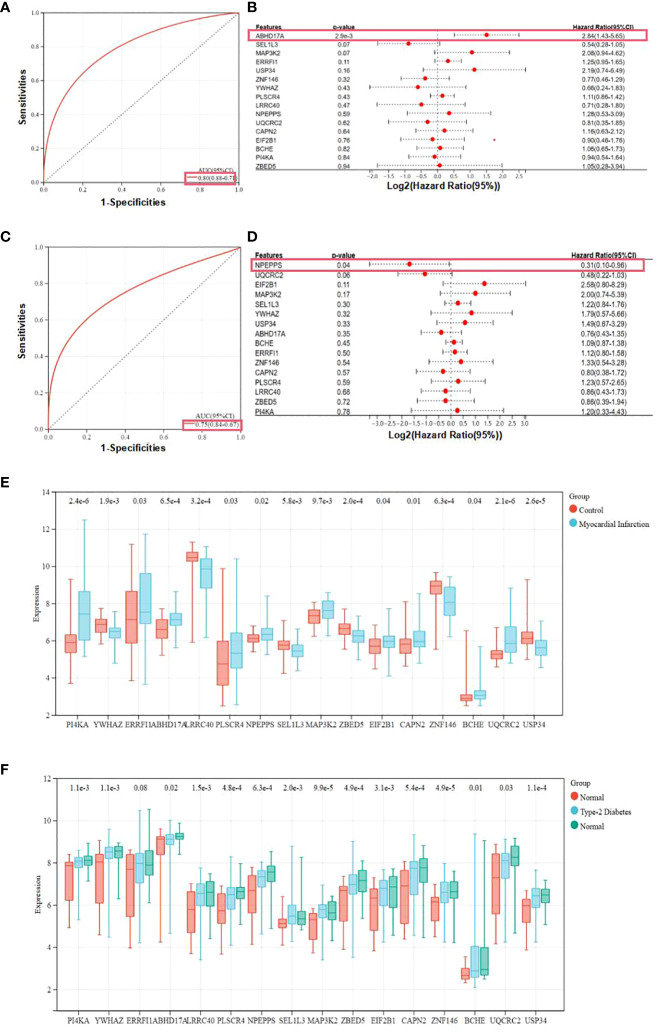
Expression and receiver operating characteristic curves values of samples and key genes from two diagnostic models. **(A)** Construction of the candidate genes-based diagnostic prediction model of CAD. **(B)** Univariate Cox regression analysis showing the HRs with 95% CIs and p values for candidate hub genes in CAD. **(C)** Construction of the candidate genes-based diagnostic prediction model of T2DM. **(D)** Univariate Cox regression analysis showing the HRs with 95% CIs and p values for candidate hub genes in T2DM. **(E)** Representation of candidate diagnostic genes in MI individuals of blood specimens in GSE66360. **(F)** Representation of candidate diagnostic genes in T2DM individuals of depots of adipose structure specimens in GSE78721.

Two predictive models incorporating candidate hub genes were developed using the logistic regression algorithm, drawing from GSE66360 and GSE78721 datasets. The prediction model built from the GSE66360 dataset exhibited strong diagnostic capabilities, with an AUC value of 0.80 (**Figure 5A**). Univariate Cox regression analysis of the expression of candidate hub genes from prediction model was conducted. The results suggested that high expression of ABHD17A (p=2.9e-3) was associated with diagnostic rates of patients with MI compared to that of healthy individuals. (**Figure 5B**). For the GSE78721 dataset, the model displayed an AUC value of 0.75 (**Figure 5C**). Univariate Cox regression analysis of the expression of candidate hub genes suggested that high expression of NPEPPS (p=0.04) was associated with diagnostic rates of patients with T2DM compared to that of healthy individuals (**Figure 5D**). When selecting pathological samples for CAD, blood samples prove to be more appropriate than adipose tissue samples, mainly because peripheral blood samples are easier to obtain *in vivo*. The results from peripheral blood specimens suggest that the GSE66360 dataset has predictive value for CAD diagnosis in practical disease treatment. Using univariate Cox regression analysis to validate candidate hub genes in the GSE66360 prediction models of MI and GSE78721 prediction models of T2DM. This validation revealed that only two hub genes, ABHD17A and NPEPPS, were noticeably up-regulated in CAD and T2DM predictive diagnostic models. Box plots showed that five genes: PI4KA, ERRFI1, LRRC40, ABHD17A and ZNF146 were noticeably expressed in MI, while the remaining eleven genes were expressed to a lesser extent such as SEL1L3, UQCRC2 and USP34. Obviously, the genes expression level of BCHE was the lowest (**Figure 5E**). In the T2DM dataset, nearly all candidate genes were highly expressed. Among them, the proportion degrees of ABHD17A and UQCRC2 were particularly pronounced, but the proportion degrees of BCHE was again the lowest (**Figure 5F**).

### The composition of immunocytes and immune infiltration

**Figure 6:**
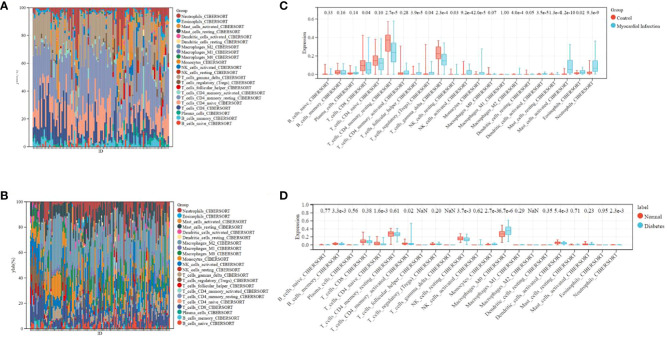
Distribution of immunocytes between diseased and normal specimens. **(A)** Relational proportion of 22 immunocytes in each specimen in GSE66360. **(B)** Relative proportion of 22 immunocytes in each specimen in GSE78721. **(C)** Variation in Immunocyte proportion expression between MI and Normal specimens. **(D)** Variation in Immunocyte proportion expression between T2DM and Normal specimens.

The microenvironment of the sample, consisting of lymphocytes, monocytes, macrophages, granulocytes, and inflammatory factors, has a significant impact on disease diagnosis and clinical therapeutic sensitivity. For this investigation, the composition of 22 types of immunocytes in different sample groups, including 49 MI cases, 50 normal cases, 105 DM cases, and 95 normal cases, were estimated using the CIBERSORT algorithm. This composition is illustrated in the histograms (**Figures 6A, B**). Comparisons were made between the immunocyte infiltration in the MI and normal groups, as well as between DM and normal specimens, and these comparisons are presented in the box plots (**Figures 6C, D**). The results suggest that in the GSE66360 dataset, there was a conspicuously higher proportion of CD8 cells (p = 0.04), CD4 memory resting T cells (p = 2.7e-5), and gamma delta T cells (p = 2.3e-4), as well as a lower proportion of activated mast cells (p = 4.2e-10) and neutrophils (p = 9.3e-9) in the normal group compared to the MI group. In the GSE78721 dataset, the normal group exhibited conspicuously higher proportions of resting NK cells (p = 3.7e-3), CD4 naive T cells (p = 1.6e-3), and activated dendritic cells (p = 5.4e-3), but lower proportions of M0 macrophages (p = 6.7e-6) contrast with the DM group. Furthermore, an analysis of the relationship between infiltration estimation and gene expression in gene modules revealed that T-cell CD4^+^ Th1 in genes ERRFI1, ZBED5, UQCRC2, ZNF146, ABHD17A, YWHAZ, and especially PLSCR4 showed a negative correlation in nearly 40 types of cancer. Many of these cancers are associated with CAD or T2DM, including UCEC, BRCA, PRAD, COAD, PAAD, and others. Modules that investigate the relationship between immune infiltrates and genomic alterations or clinical outcomes in TCGA are displayed in **Supplementary Figure 3**.

### Study of immune cells in CAD, T2DM, and healthy individuals

**Figure 7:**
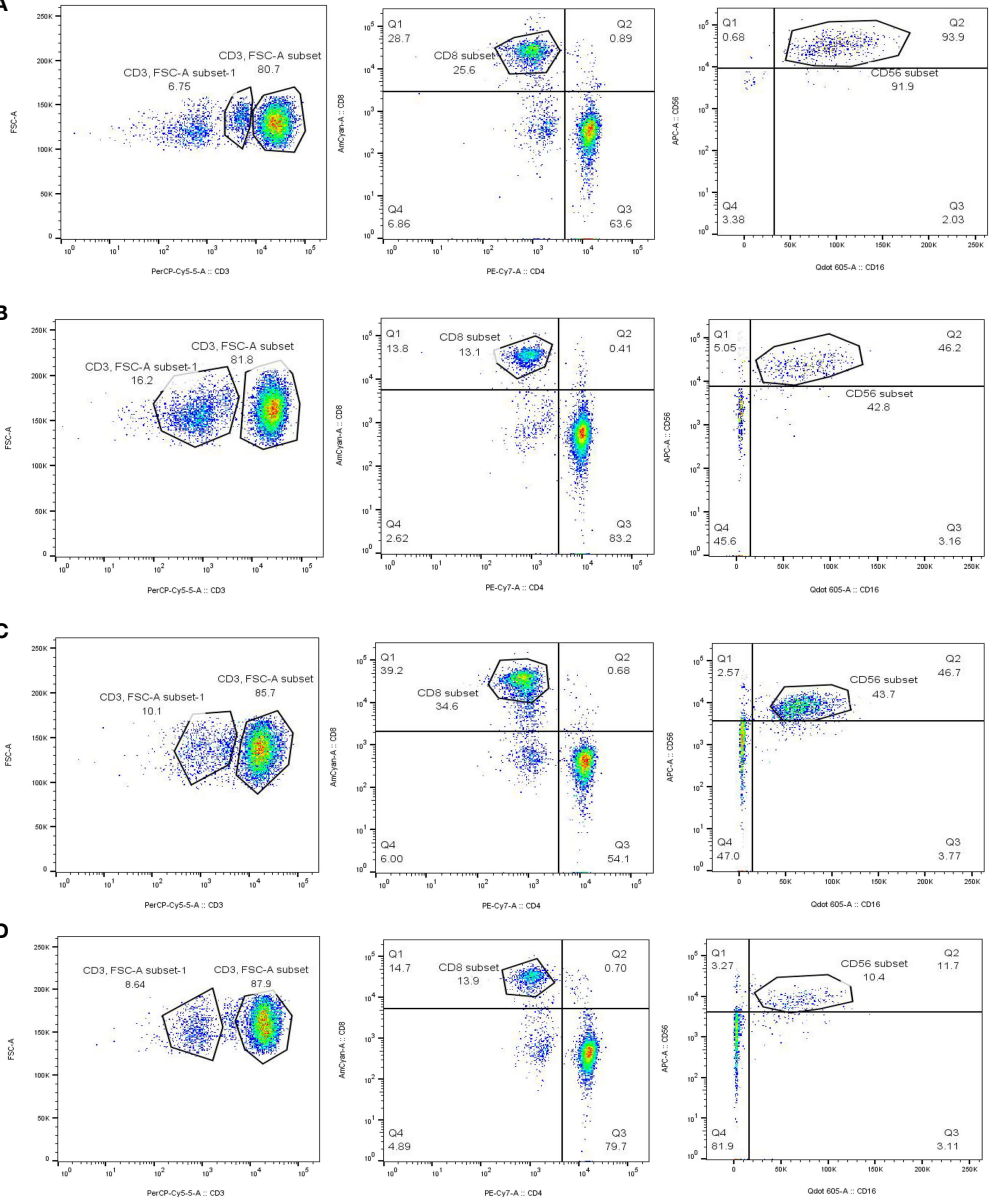
Gating strategy for immune cells of CAD, T2DM patients and healthy people. **(A)** The blood count of CD8^+^T and NK cells in healthy people. **(B)** The blood count of CD8^+^ T and NK cells in CAD patients. **(C)** The blood count of CD8^+^ T and NK cells in T2DM patients. **(D)** The blood count of CD8^+^ T and NK cells in CAD combined with T2DM individuals.

Following the bioinformatics analysis of immune cell expression in CAD and T2DM, clinical samples were collected for the clinical validation of immune cells using flow cytometry. Prior to the flow cytometry analysis, suitable gating strategies were utilized to identify cells with live/dead staining in CD8 T cells and NK cells using fresh cells (freshly isolated from peripheral blood), as some biomarkers such as CD16 may undergo downregulation or detachment after thawing. Blood counts of CD8^+^ T and NK cell lymphocytes were detected by flow cytometry in 38 patients from the First Affiliated Hospital of Anhui University of Chinese Medicine, categorized as those with CAD, T2DM, CAD Combined with T2DM, and 9 healthy subjects (**Tables 2**–**4**). The CD8^+^T and NK cells in CAD, CAD Combined with T2DM, and normal venous blood were observed through flow cytometry (**Figures 7A–D**). Combined with **Tables 3** and **4**, the results demonstrated that noticeably higher percentages of CD8^+^T cells are typically present in healthy subjects (**Figure 7A**) compared to those with CAD (**Figure 7B**), and higher levels of NK^bright^ cells in healthy subjects compared to those with CAD or T2DM (**Figure 7C**). It was observed that healthy subjects typically have conspicuously higher proportions of NK^bright^ cells than those with CAD combined with T2DM (**Figure 7D**).

**Table 2:** The demographics and clinical characteristics of individuals.

	NOR Control (n=9)		CAD (n=13)	T2DM (n=11)	CAD-T2DM (n=14)
**Female/Male**	4/5		7/6	6/5	7/7
**Mean age**	62.8 ± 4.5		61.8 ± 4.6	64.1 ± 4.4	62.7 ± 5.0
**BMI**	22.5 ± 2.5		24.3 ± 2.1	25.4 ± 3.0	23.0 ± 2.6
**HbA1c, %**	5.32 ± 0.55		5.29 ± 0.44	8.90 ± 1.57	7.49 ± 1.18
**TC, mmol/L**	5.18 ± 1.09		4.91 ± 0.73	6.21 ± 1.05	6.16 ± 1.21
**TG, mmol/L**	1.81 ± 0.68		1.61 ± 0.79	3.3 ± 0.96	3.17 ± 1.38
**LDL-c, mmol/L**	2.78 ± 0.96		2.65 ± 0.94	3.03 ± 1.27	2.89 ± 1.31

**Table 3:** The levels of CD8^+^ T and NK cells in patients.

Group	CD8^+^T Cell		NK Cell	
	High	low	CD56^bright^	CD56^dim^
Normal	7(77.8%)	2(22.2%)	6(66.7%)	3(33.3%)
CAD	5(38.5%)	8(61.5%)	4(31.0%)	9(60.0%)
T2DM	8(72.7%)	3(27.3%)	4(36.4%)	7(63.3%)
CAD & T2DM	4(28.6%)	10(71.4%)	3(21.4%)	11(78.6%)

**Table 4:** Changes in CD8^+^ T and NK cell levels in peripheral blood (%).

Group	n	CD8^+^T Cell	NK^bright ^Cell
Normal	9	26.77±2.96	89.02±2.87
CAD	13	14.71±4.22	40.81±3.98
T2DM	11	31.45±3.76	42.05±2.80
CAD-T2DM	14	11.64±4.33	17.87±6.08

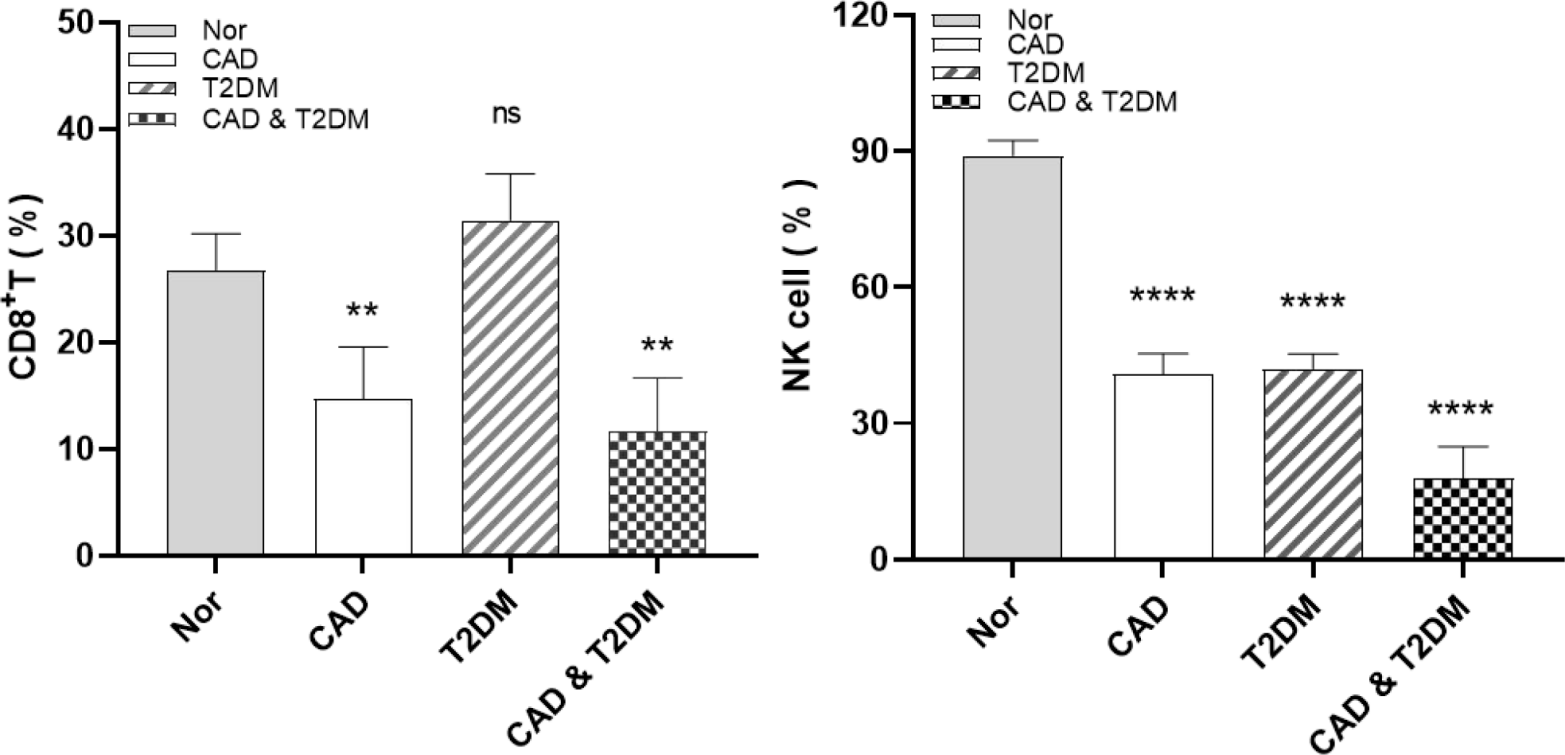

**p<0.01; ****P<0.0001; ns, no significance.

## Discussion

Early diagnosis of CAD combined with T2DM is challenging due to its complicated etiology and risk factors. Therefore, it is crucial to develop new diagnostic models to identify the drivers of CAD associated with T2DM. In this study, bioinformatics research identified 16 candidate hub genes associated with gene co-expression relationships and up-regulated genes in CAD and T2DM. NPEPPS and ABHD17A were identified as key genes in CAD combined with T2DM patients and were highly associated with the diagnosis of CAD and T2DM. Subsequently, we highlighted the important roles of CD8^+^ T cells and NK cells in the pathogenesis of these two diseases using CIBERSORT and immune infiltration, suggesting that they may be viable targets for diagnosis and therapeutic intervention. The discovery of key diagnostic genes and significant changes in immune cells, specifically CD8^+^ cells and NK cells, in CAD combined with T2DM provides new insights into potential targets for diagnostic and therapeutic interventions.

Further bioinformatics analysis revealed that these 16 candidate hub genes were associated with various protein biological processes. The key diagnostic gene, ABHD17A, is associated with the biological process of protein export and ubiquitin-mediated proteolysis. ABHD17A has significant catalytic activity to play a key role in membrane localization, and promotes N-Ras deacylation, leading to changes in the subcellular localization of N-Ras. Additionally, it promotes palmitate turnover on proteins such as PSD95 and N-Ras, which are important processes that control protein localization and signal transduction (34). The other diagnostic key gene, aminopeptidase (NPEPPS) is an important zinc metallopeptidase belonging to the oxytocinase subfamily of the M1 aminopeptidase family (35, 36). It contributes to the machining of the proteosome-acquired peptide pool, following closely behind pruning of antigen peptides by ERAP1 and ERAP2 for emergence on major histocompatibility complex (MHC) class I molecules (35, 37). Several GWAS analysis have presented relevances of these NPEPPS with multifarious immunity-induced disorders for instance inflammatory bowel disease, and diabetes mellitus, the genetic interactions between some aminopeptidases and HLA class I loci are closely related to these diseases (38–43). In this study, multiple bioinformatic analyses have established that CAD and T2DM are tightly associated through the hub genes ABHD17A and NPEPPS. Unfortunately, these analyses and subsequent validation, by applying clinical samples, are not sufficient to elucidate whether ABHD17A and NPEPPS are a cause or a consequence of T2DM or CAD. Both ABHD17A and NPEPPS are related to cell metabolism and play important roles in phosphatidylinositol metabolism, which may be significant in promoting T2DM progression. In addition, NPEPPS is closely associated with various autoimmune diseases. Despite these findings, since T2DM is a long-term chronic metabolism disease, it affects the metabolic changes in the body, which will affect the function of the immune system, and the progression of CAD, especially the occurrence of MI, is closely related to the abnormal function of the immune system. Therefore, when focusing on ABHD17A and NPEPPS in CD8^+^ T cells and NK cells, we tend to believe that the abnormalities of these hub genes in these immune cells are caused by long-term metabolic changes caused by T2DM. Subsequently, these abnormalities in immune cells caused by T2DM contribute to the progression of CAD and increase the risk of MI. These are considerations based on disease characteristics and known hub gene functions. Additional research is required to clarify the pathogenic mechanisms of ABHD17A and NPEPPS in CAD and T2DM and establish specific causal relationships.

This study found that CD8^+^ T cell ratios was higher in healthy individuals than in CAD patients and CAD complicated with T2DM patients. Notably, the proportion of NK^bright^ cells in healthy individuals is usually significantly higher than CAD or T2DM patients. Recent studies revealed that CAD patients have a high number of CD8^+^ T cells expressing CD56 or CD57, which exhibited typical pro-inflammatory features (44–46). Dilated CD8 IL-6Rα^+^ low T cells were associated with increased incidence of failure, cytotoxic CD8CD57 T cells, and elevated IL-6 levels. The expression of IL-6Rα by human CD8^+^ T cells has been considered to define a distinct T cell subset that produces Th2 cytokines (47). Simultaneously, In patients with CAD, NK cell apoptosis, a key factor in initiating and regulating the immune response, is reduced (48). Furthermore, a potential negative impact on immunomodulatory defenses during the development of atherosclerosis may result from the sustained loss of NK cells (26, 49, 50). NK cells from the patients had suppressed both TNF-α secretion and particle capability as evidenced by CD107a reflection (51).

T2DM is known to be a major risk factor for CAD. Among T2DM patients, CAD is more likely to be a complicated disorder characterized by small, extensive, calcified, multivessel disease (MVD) and often requires coronary revascularization apart from definitive medical treatment to control angina pectoris (52). Research has shown that insulin resistance, hyperinsulinemia, and vascular calcification are common complications in diabetes patients (53). Promotive factors, such as diabetes-induced ROS overexpression, secretion of inflammatory factors, improved conversion rate of aldose reductase (AKR1B1) basement (54), and activation of protein kinase C β, δ, and θ, can accelerate the transformation of stable plaque into unstable plaque or plaque rupture (51, 52), which subsequently leads to thrombosis and the manifestation of adverse coronary events (51).

Inevitably, the above study has limitations. Diagnostic models were constructed for the diagnostic prediction of patients with CAD combined with T2DM based on retrospective data from the GEO database. The model is based on 16 candidate hub genes. Prospective data is needed to validate the clinical application value of the model. Further investigation is needed to determine the specific mechanism of action of ABHD17A and NPEPPS in CD8^+^ and NK cells.

## Materials and methods

### Study design and data collection

The NCBI Gene Expression Comprehensive Public Database (GEO) provides source support for data collection and subsequent analysis. GSE66360 annotated HG-133U from GPL570 in peripheral blood_PLUS_2 microarray measurement of gene expression, which included 49 myocardial infarction groups and 50 healthy cohorts. GSE78721 was annotated by GPL15207 from different adipose depots (thigh, visceral and subcutaneous) of patients suffering from type 2 diabetes.

### WGCNA of T2DM and CAD

The Sangerbox 3.0 software package, which includes “WGCNA,” was used to produce a gene co-expression network to explore the co-expression relevances between genes in the sample and the relevance of genes and their expressions. This process required a Pearson correlation matrix and an average linkage method for all pairs of genes. A weighted adjacency matrix was constructed using the power function A_Mn = |C_Mn|^β. When the soft threshold is 16.087, the R^2 has a significant improvement, reaching 0.9. At this point, the network has already followed a scale-free distribution. After choosing a power of 16.087, the contiguity was changed into a Topological Overlap Matrix (TOM). This matrix determined the network relevancy of genes, considered as the summation of their contiguity to all others in the network relative to the gene proportion, and calculated the associated diversity (1-TOM). To group genes with comparable description characteristics into gene modules, mean integration collecting was conducted according to the TOM-based diversity estimation. The minimal size for the gene dendrogram (tree) was set at 30. For further module analysis, we evaluated the module’s own genetic diversity, picked a cut-off for the module dendrogram, and combined certain modules. Each module was represented by a different color. The gene expression profile of each module was expressed by three factors: module eigengene (ME), module membership (MM), and gene significance (GS). MEs were applied to estimate the relevance between different modules and phenotypes. Module membership (MM) indicates the correlation between a gene and its corresponding module. Gene significance (GS) represented the relationship between a gene and phenotype, and was determined by the log10 transformation of the P value in the linear regression between gene expression and phenotype.

### Identification of differentially expressed genes

Perform expression analysis of diverse genes on CAD (GSE66360) and T2DM (GSE78721) samples using the “limma” software package from the online website Sangerbox 3.0. P_ Genes with adj < 0.05 and multiple variation (FC) | > 1.5 were regarded as distinctively described genes. Create heat maps and volcanic maps of differentially expressed genes using the “pheatmap” and “ggplot2” software packages. Use the online Venn chart tool to obtain their common 16 DEGs.

### Functional enrichment analysis

The online website sangerbox carries out GO analysis and KEGG analysis, in which GO analysis includes BP (Biological Process), MF (Molecular function), CC (Cellular configuration), cardiovascular disease samples and diabetes samples, as well as hub gene enrichment investigation. GSEA was served as reveal the respective functions of central genes. Using the Gene Ontology footnote in R program procedure (edition 3.1.0) for the technical support, enrichment analysis of gene set functions was conducted, genes are plotted to the backdrop set, Enrichment investigation was conducted enacting R program clustering archive (edition 3.14.3) to derive gene set enrichment outcomes. Setting the minimum gene set to 5 and the maximum gene set to 5000, with a P value of < 0.05 and an FDR value of < 0.25, is considered statistically relevantly.

### Production of receiver operating characteristic curves and description of hub genes in samples

We operated R program pROC (edition 1.15.0.1) to conduct ROC estimation to acquire AUC. Specifically, we obtained the CAD and T2DM gene expression of patients, used the ROC function of pROC to conduct ROC analysis at 365 time points, and used the ci ability of pROC to estimate AUC and confidence intermission to acquire the final AUC outcomes.

### Gene set enrichment investigation

We assembled GSEA (http://software.broadinstitute.org/gsea/index.jsp). The web page obtained GSEA program (edition 3.0) and separated the models into two series according to disease types. The various immune gene samples were collected from the immunologic signature gene sets (http://www.gsea-msigdb.org/gsea/downloads.jsp). The kegg characters subset was downloaded to estimate relative pathways and molecular mechanisms of action. According to gene reflection profiling and phenotype subsets, set the minimal genomes and utmost genomes, and collect samples again, P numerial number of < 0.05 and an FDR of < 0.25 were consistent statistically significance relevant.

### Immunocyte infiltration and level in diverse cancer types

CIBERSORT is used to evaluate the infiltration of immunocytes in the human microenvironment. This reagent includes 547 biomarkers and 22 humanity immunocytes, comprising lymphocytes (T cells and B cells), Monocyte, neutrophils, macrophages, etc. The figures were Presented as stacked bar charts through the online platform Sangerbox (http://vip.sangerbox.com/home.html). Various cancer types in Immune infiltration level were presented as heatmap, and figures will indicate the fineness-restructured spearman’s rho pass through assorted cancer categories through the online platform (http://timer.comp-genomics.org/timer/).

### Diagnosis standard for CAD and T2DM

According to the diagnostic criteria by the American Diabetes Association and International Society of Hypertension, our study employed an case–control design, which included the selection of the 38 most rapidly progressing CAD, T2DM and CAD combined with T2DM cases from the clinical study. CAD was defined as: (1)Male patients aged over 40 and female patients aged 45 and above; (2)Coronary artery stenosis≥50% based on CAG or CCTA examination; (3)Symptoms such as chest tightness or chest pain undergo a comprehensive evaluation on admission. T2DM was defined as: (1)FPG ≥ 7.0mmol/L; (2)PBG ≥11.1mmol/L; (3)HbA1c≥6.5%. Controls included participants with no evidence of T2DM and no evidence of CAD by 65 years of age.

### Detection of immunocytes infiltration in patients and healthy cohorts

We screened 38 patients with CAD or T2DM from the Department of Cardiology of the First Affiliated Hospital of Anhui University of Chinese Medicine, as well as 9 eligible healthy volunteers, and conducted flow cytometry immune examinations. Firstly, blood is taken from the human body to prepare samples, prepare cell suspension, count cells, use EP (1.5ml) tube for sub packaging, level rotor 800g, 4°C centrifugation. After completion, use a Pap pipette to remove the middle white membrane layer, add 5ml PBS for resuspension, level rotor 250g, 4°C centrifugation and add appropriate fluorescence-labelled antibodies. Mix well, avoid light and incubate at 4°C for 30 minutes. After completion, add 1ml PBS to wash twice and finally resuspend with 200ul PBS. Then adjust the laser light source, detector and flow rate, and load the prepared cell sample into the flow cytometer (BD LSR Fortessa) to detect the expression of CD8^+^T and NK cells, collect and count the proportion of CD8^+^T and NK cells in CAD, T2DM and healthy samples, and obtain the final results.

### Numerical statement manipulation

The data processing were manipulated in R program and loads of online websites. The selection and use of data in the article are based on the criterion of significance P < 0.05.

## Data availability statement

The original contributions presented in the study are included in the article/**Supplementary Material**. Further inquiries can be directed to the corresponding authors.

## Ethics statement

All human blood samples were collected with informed consent from patients, and all related procedures were performed with the approval of ethics boards of the The First Affiliated Hospital of Anhui University of Chinese Medicine.

## Author contributions

CD: Data curation, Methodology, Software, Writing – original draft, Supervision. DW: Data curation, Methodology, Writing – original draft, Supervision. QT: Supervision, Writing – original draft. ZL: Formal analysis, Writing – review & editing. PZ: Investigation, Writing – original draft. YW: Writing – original draft, Conceptualization, Data curation, Visualization. MH: Investigation, Validation, Writing – original draft. SC: Validation, Conceptualization, Writing – original draft. WQ: Data curation, Supervision, Writing – original draft. LZ: Writing – review & editing. HY: Funding acquisition, Visualization, Writing – review & editing.
