# Supplementary figures and images for: CD8+ T and NK cells characterized by upregulation of NPEPPS and ABHD17A are associated with the co-occurrence of type 2 diabetes and coronary artery disease
Source: Front Immunol. 2024 Feb 23;15:1267963. doi: 10.3389/fimmu.2024.1267963 (PMC10921359; doi:10.3389/fimmu.2024.1267963)

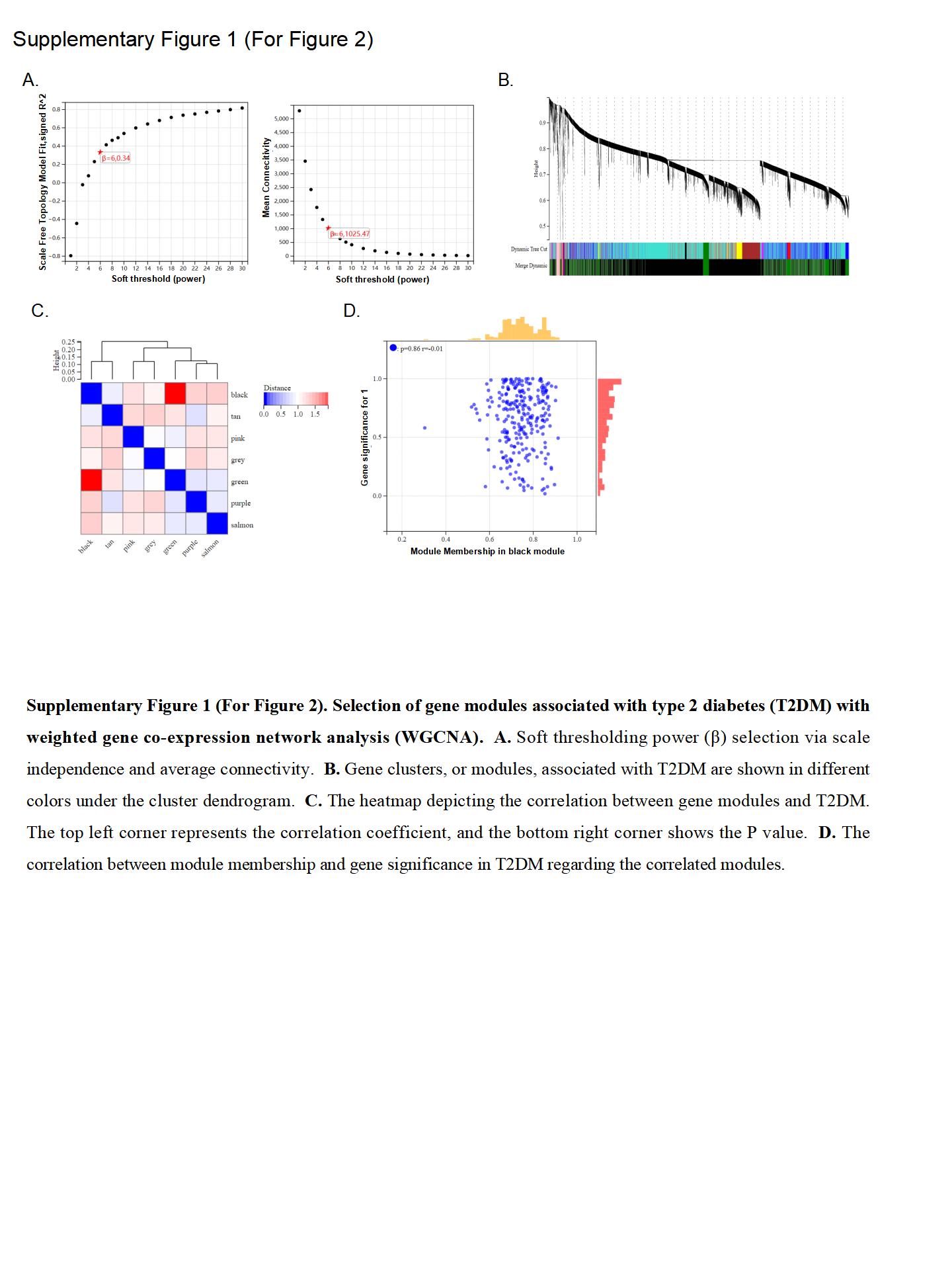

Supplement: Supplementary file 1 [file Image_1.jpeg]

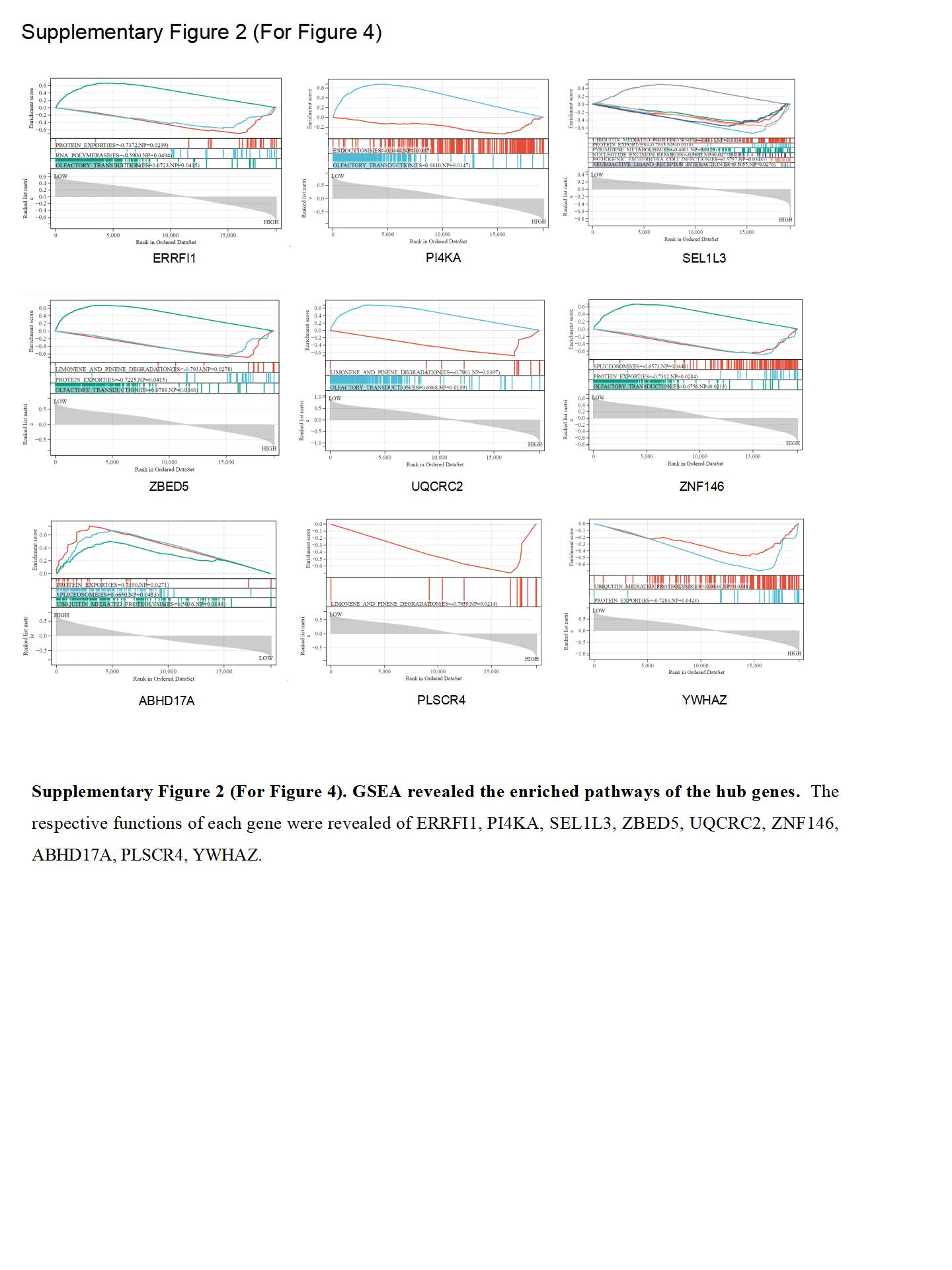

Supplement: Supplementary file 2 [file Image_2.jpeg]

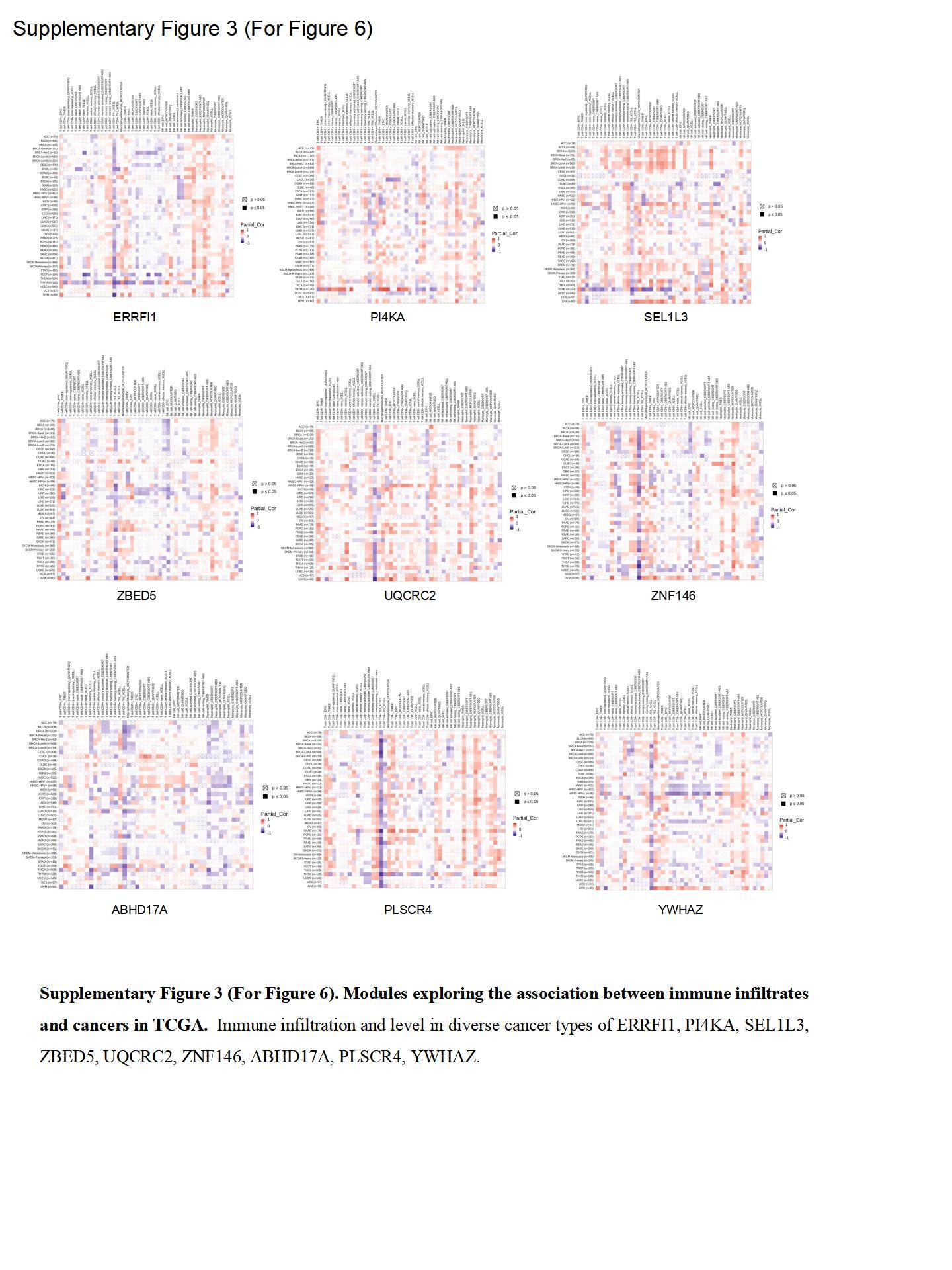

Supplement: Supplementary file 3 [file Image_3.jpeg]
